# Alport syndrome complicated with IgA nephropathy: a case report

**DOI:** 10.3389/fmed.2026.1739845

**Published:** 2026-02-16

**Authors:** Jing Sun, Fangfang Yu

**Affiliations:** 1 Department of Nephropathy Rheumatology and Immunology, The People’s Hospital of QianNan, Duyun, China; 2 Department of Nephrology, Guizhou Provincial People’s Hospital, Guiyang, China

**Keywords:** Alport syndrome, gene detection, IgA nephropathy, proteinuria, renal biopsy

## Abstract

Alport syndrome (AS) and immunoglobulin A (IgA) nephropathy (IgAN) are distinct renal disorders characterized by hematuria and proteinuria. AS is a rare hereditary condition caused by mutations in genes encoding collagen IV *α*-chains, leading to abnormalities of the glomerular basement membrane. However, IgAN is an autoimmune disorder characterized by glycosylation defects in IgA1, leading to dysfunction of glomerular filtration. Approximately 15% of IgAN cases exhibit familial clustering, and some may harbor gene mutations associated with AS. A 29-year-old woman with no family history of nephropathy presented with hematuria and proteinuria. The renal biopsy revealed foamy interstitial cells. Electron microscopy indicated a torn basement membrane, indicating a diagnosis of concurrent AS and mild mesangial proliferative IgAN. Genetic testing confirmed a mutation consistent with X-linked AS. Moreover, she exhibited high-frequency hearing loss in her left ear. Initial treatment included angiotensin receptor blockers and sodium-glucose co-transporter-2 inhibitors. Corticosteroids and mycophenolate mofetil were introduced prior to genetic testing, although their efficacy was limited. Despite the co-occurrence of multiple nephropathies, this case suggests the usefulness of renal biopsy and genetic testing for accurately diagnosing kidney disease, which can help initiate appropriate treatments early. The treatment regimen did not provide significant benefits in this patient with concurrent AS and IgAN. This emphasizes the need for targeted diagnostics and personalized therapeutic approaches.

## Introduction

Alport syndrome (AS) is a rare hereditary disease with a prevalence of 1/5000–1/53,000 (1). It is caused by mutations in collagen type IV alpha 3–5 (COL4A3–COL4A5) genes, leading to abnormal type IV collagen structure and consequently lesions of the glomerular basement membrane (GBM). Based on the mode of inheritance, AS can be classified into three forms: X-linked (XLAS), autosomal recessive (ARAS), and autosomal dominant (ADAS) (1), Mutations in COL4A3–COL4A5 gene disrupt type IV collagen α3-5 chains, which affect GBM, eyes, and ears, clinically manifesting as hematuria, proteinuria, sensorineural hearing loss, and retinopathy.

IgA nephropathy (IgAN) is the most common glomerular disease in China. It arises from the deposition of IgA1-containing immune complexes in the glomerular mesangium, triggering immune-mediated glomerular injury. Although the pathogenesis of IgAN remains incompletely understood, the widely accepted “four-hit” hypothesis proposes a sequence of events involving: ① elevated levels of galactose-deficient IgA1 (Gd-IgA1), ②production of autoantibodies against Gd-IgA1, ③ formation of pathogenic immune complexes, and ④ their deposition in the mesangium, ultimately leading to glomerular damage. It has also been argued by some scholars that physical stress on glomerular capillaries may result in rupture of the glomerular basement membrane (GBM), causing capillary loop prolapse and podocyte detachment, which contributes to proteinuria (2).

The coexistence of AS and IgAN is exceptionally uncommon (3). Although both are kidney diseases with distinct etiologies—AS being caused by pathogenic variants in COL4A3–5genes and IgAN primarily driven by immune-mediated mechanisms—they share overlapping clinical features, particularly hematuria and proteinuria, which often complicate differential diagnosis (4). Notably, emerging evidence suggests that familial IgAN may, in some instances, involve mutations in AS-associated collagen IV genes, indicating a potential genetic intersection between these two entities. Here, we report a case of concurrent AS and IgAN, providing novel clinicopathological evidence supporting their possible coexistence.

## Case presentation

A 29-year-old female patient was admitted to the hospital on March 11, 2023, due to proteinuria. This condition was first identified during a pregnancy test at 13 weeks of gestation. Multiple tests throughout her pregnancy consistently revealed a urinary protein level of 3+. She underwent a renal biopsy following childbirth. She exhibited no symptoms such as rash, joint pain, dry mouth, dry eyes, or blurred vision. Her parents were healthy with no history of kidney disease.

**Figure 1:**
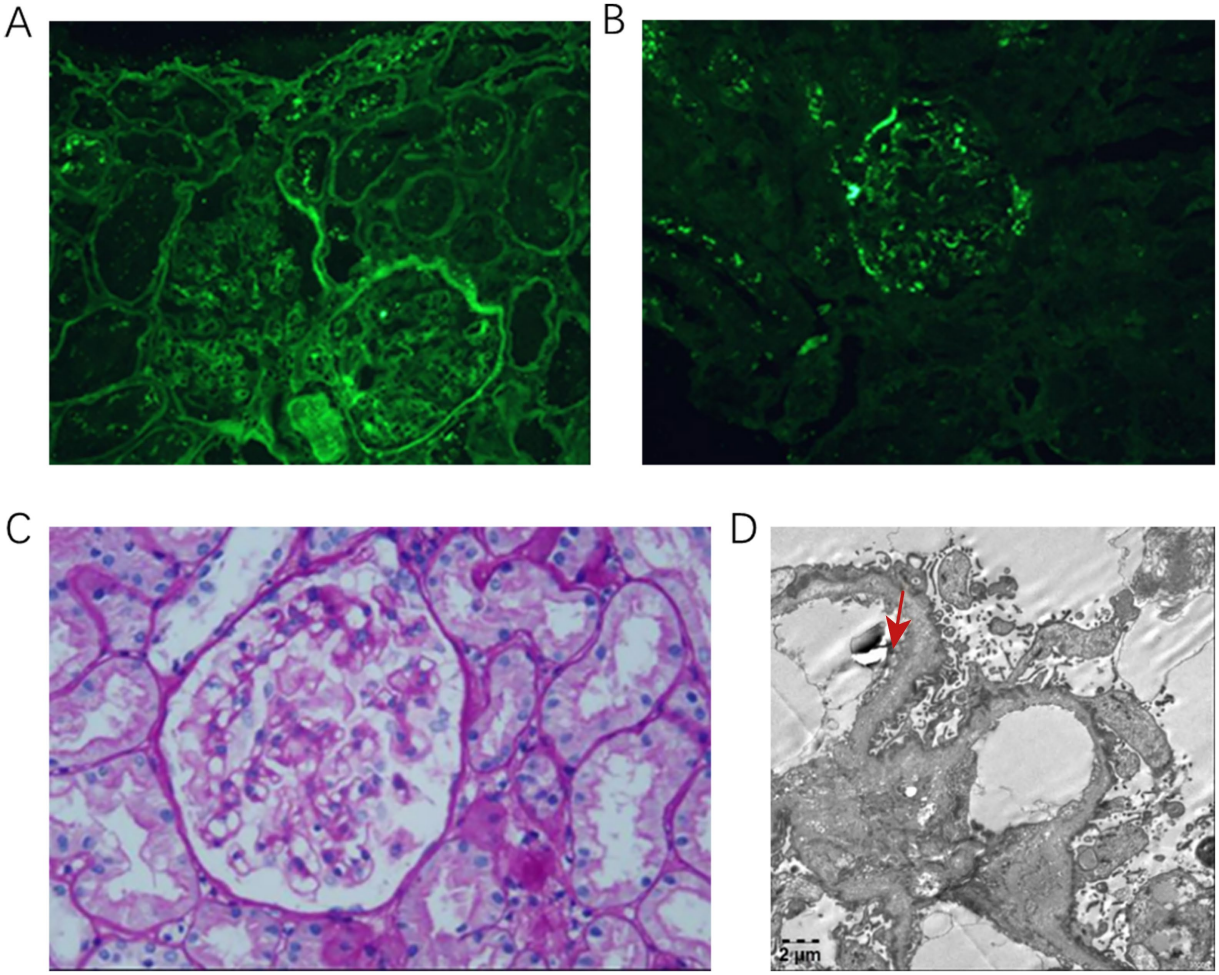
**(A,B)** IgA deposits in a punctate pattern along the mesangial area. **(C)** Light microscopy: mild proliferation of glomerular mesangial cells and matrix. **(D)** Electron microscopy: capillary basement membrane: the thickness of the basement membrane varied, with a thickness of about 140–600 nm. The dense layer of the basement membrane thickened, and some parts exhibited tearing and spider-like patterns.

Upon admission, her blood pressure was 122/72 mm Hg with no lower limb edema. Laboratory tests demonstrated normal complements, autoantibodies, blood count, and organ function. Urinalysis revealed 2.14 g/24-h proteinuria, low serum albumin (32.2 g/L), and hematuria. Renal biopsy showed IgA (+++), IgM (+), C3 (++), and tubular reabsorption droplets (+), with other markers negative. Microscopic examination revealed 15 glomeruli and one instance of segmental glomerulosclerosis (Figures 1A,B). The glomerular mesangial cells and stroma exhibited slight proliferation, with red blood cell deposition in the mesangial area and open capillary loops (Figure 1C).

Renal tubular epithelial cells displayed vacuoles and granular degeneration, with focal tubular atrophy and interstitial infiltration of inflammatory cells (no fibrosis). Glomeruli had capillary vacuolar degeneration; capsule cells were vacuolated without proliferation.

The thickness of GBM varied, measuring approximately 140–600 nm, with a thickened dense layer. Some areas appeared torn and cobweb-like (Figure 1D). The epithelial cells were swollen and vacuolated, with diffuse foot process fusion. Mesangial cells and matrix proliferation were observed. Electron microscopy revealed electron-dense deposits in the mesangial region and vacuolar degeneration in the renal tubular epithelial cells within the renal tubule–interstitial areas. No unique lesions were identified in the renal interstitium, although red blood cells were observed in the capillary lumens, along with foamy interstitial cells. GBM tears were noted, strongly suggesting AS. The patient’s condition was consistent with mild mesangial proliferative IgAN, classified as Grade II according to the Lee classification system, and M1 E0 S1 T0 C0 according to the Oxford classification criteria for IgAN.

**Figure 2:**
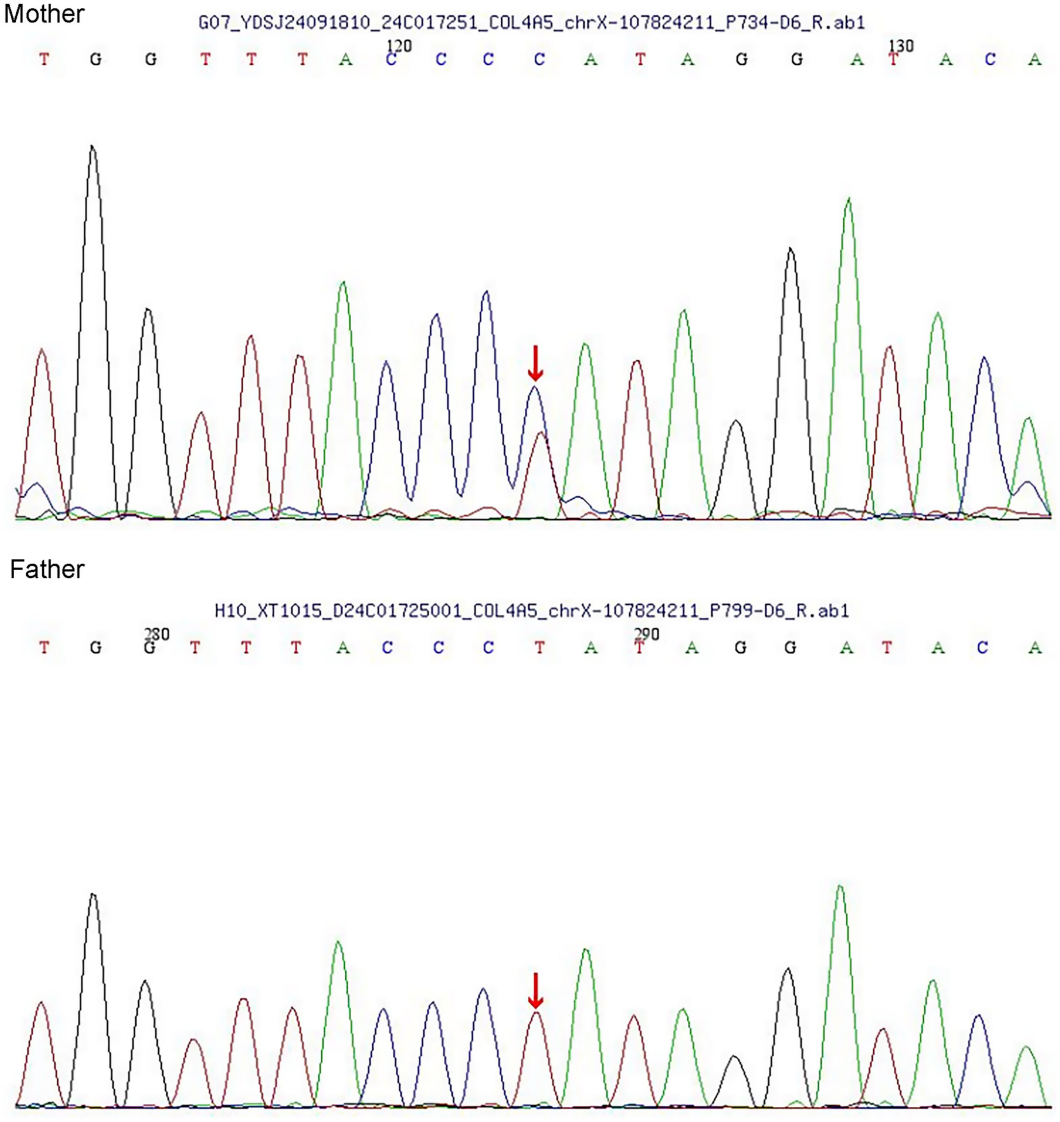
Sanger sequencing of the parents.

In terms of treatment, the patient received continuous therapy with an angiotensin II receptor blocker (ARB) and a sodium-glucose cotransporter-2 (SGLT2) inhibitor throughout the disease course. Prior to genetic confirmation, a short course of oral prednisone acetate and mycophenolate mofetil was added empirically. The prednisone regimen started at 24 mg daily and was tapered by 4 mg every two weeks, while MMF was administered at a dose of 1.5 g per day. At the 3-month follow-up,the patient’s proteinuria markedly decreased but did not achieve partial remission, with a 24-h urinary protein excretion of 1.3 g/24 h—still exceeding the 1 g/24 h. July 2024 whole-exome sequencing revealed a heterozygous COL4A5 mutation (c.892-2A > G). The patient’s parents were tested in October,and both had normal urinalysis with no proteinuria or hematuria. Sanger sequencing confirmed a heterozygous mutation in the mother, whereas the father was mutation-negative (Figure 2). This variant was classified as pathogenic (ACMG Class 5). Based on these findings, the final diagnosis was AS complicated by IgAN. The patient was treated with oral mycophenolate mofetil, prednisone acetate, SGLT2i and irbesartan. The dosages of mycophenolate mofetil and prednisone acetate were gradually reduced and eventually discontinued 6 months later. At that time, the patient was taking oral losartan, with proteinuria measuring 3 + and a 24-h urine protein level of 3.6 g. Renal function remained normal, with a serum creatinine level of 53 μmol/L and an estimated glomerular filtration rate (eGFR) of 124.5 mL/min/1.73 m^2^. The patient declined further immunosuppressive therapy, and proteinuria persisted.

## Discussion

This case reveals coexisting IgAN and AS with hematuria, proteinuria, and renal impairment but distinct etiologies/pathologies. It emphasizes the importance of accurate diagnosis using histopathology, electron microscopy, and genetic testing, noting that type IV collagen immunofluorescence could not be performed because of financial constraints.

Familial IgAN is one of the types of IgAN inherited in the X-linked recessive or autosomal dominant mode (5). Family analysis and pathogenic gene detection of familial IgAN among Han people in northern China revealed that the mutation C.653 g > A (P.R218q) in the *INF2* gene was pathogenic (6). Liu et al. (7) screened four candidate genes (*MYCT1*, *CARD8*, *ZNF543*, and *DEFA4*), but their functions need further verification. Stapleton et al. (8) sequenced the exons of 10 IgA families in Ireland and found 3 mutations in *COL4A3*, *COL4A5*, and *LMX1B*, among which *COL4A5* p. (Gly1143Ser) was identified as a harmful mutation. Gene mutations also play a role in AS progression, which is divided into XLAS, ARAS, and ADAS. XLAS is caused by mutations in the *COL4A5* gene, whereas ARAS is caused by homozygous or compound heterozygous mutations in the *COL4A3* and *COL4A4* genes. ADAS is caused by heterozygous mutations in the *COL4A3* or *COL4A4* gene (9). The severity of the clinical phenotype caused by COL4A5 mutations is related to the patient’s sex, gene mutation type, and mutation location (1). Female patients with XLAS have different phenotypes due to the Lyon phenomenon of X chromosome inactivation. Also, the severity is lighter in female patients than in male patients, with an incidence of end-stage renal disease of 10% in XLAS women but 90% in XLAS men (10). Up to 1,000 mutations were identified in *COL4A5*, with missense mutations accounting for about 50%. Large fragment deletion/recombination/insertion, nonsense mutations, shear mutations, and frameshift mutations, especially truncated mutations, are more serious in kidney and extra-kidney manifestations (1). Patients with mutations in exons 23–51 have more serious clinical manifestations than those with mutations in exons 1–20, enter renal failure earlier, and are more prone to renal abnormalities (1).

Both AS and IgAN can manifest as hematuria and proteinuria, making differential diagnosis challenging. Reports show patients initially diagnosed with IgAN who were later found to have AS on genetic or ultrastructural analysis, and vice versa. Sometimes, both conditions may co-occur in the same family or individual, leading to diagnostic ambiguity and potentially altered therapeutic approaches (4, 11). A genetically weakened GBM in AS may be more susceptible to the deleterious effects of immune complex deposition observed in IgAN. A defective matrix can facilitate the abnormal accumulation and persistence of immune complexes, thereby exacerbating inflammation and leading to a synergistic acceleration of glomerular injury (11, 12). The ongoing tissue damage and chronic inflammation in AS can result in sustained activation of innate immune pathways, as observed by upregulation of pro-inflammatory signaling in animal models. If IgAN is also present, this pro-inflammatory milieu can further drive immune complex–mediated injury, creating a feedback loop of damage (12, 13). The presence of both a structural genetic defect and an overlying immune-mediated process can synergistically accelerate the progression to end-stage kidney disease compared with either disorder in isolation. The underlying genetic fragility may diminish the kidney’s resilience to immune attacks, and the immune process may, in turn, worsen the structural injury (4, 11).

The coexistence of these conditions complicates management. Immunosuppression—often used in IgAN—may be less effective or carry increased risks in patients with AS, who primarily have structural rather than inflammatory disease. Therefore, personalized treatment strategies and a comprehensive diagnostic workup, including genetic testing and electron microscopy, are essential in these cases (4, 11). The treatment for this patient primarily targeted IgAN due to the absence of initial genetic testing. The regimen included RAAS inhibitors, SGLT2 inhibitors, mycophenolate mofetil, and low-dose glucocorticoids (14). Although angiotensin-converting enzyme inhibitors (ACEIs) or ARBs are standard therapies for both AS and IgAN, their efficacy is modest,and combining them is contraindicated due to safety concerns(the risk of hyperkaliemia). Emerging treatments, such as sparsentan (a dual endothelin-A and angiotensin II receptor antagonist), show promise in reducing proteinuria and slowing disease progression (9). Sparsentan can reduce proteinuria and improve hearing (15). The drug was approved for treating IgAN in the United States of America (16). However, its curative effect in patients with AS is unclear. A large randomized controlled trial in China confirmed that mycophenolate mofetil combined with low-dose glucocorticoid can effectively treat IgAN without any adverse reactions (17). As a result, MMF is widely used in clinical practice in China for IgAN; however, current evidence supports its use only as a steroid-sparing agent—and specifically in Chinese patients. In accordance with this approach, our patient was treated with MMF in combination with low-dose glucocorticoids. Hydroxychloroquine, which modulates immune responses, has also exhibited potential in reducing proteinuria in both conditions. Hydroxychloroquine suppresses the immune system by inhibiting the production of Toll-like receptors and cytokines, thereby reducing proteinuria in patients with AS (9). It can also be used to treat IgAN (18). Still, many treatments are under study. Some drugs have advanced to clinical trials, including molecular chaperones, stem cell therapy, and gene therapy. Research advances on innovative therapeutic approaches for this condition are impressive and may bring hope to the millions of patients affected.

A previous case of a 27-year-old male patient with coexisting IgAN and AS was reported in the United States of America in 2021 (3). Another patient with AS-like symptoms was finally diagnosed with IgAN and Fabry disease; the case highlighted the need for early diagnosis and intervention (19). The patient had a strong family history of kidney disease. The patient had a non-nephrotic 24-h urinary protein level not requiring treatment at the time the report was published.

Despite the co-occurrence of multiple nephropathies, this case underscores the critical role of renal biopsy and genetic testing in achieving an precise diagnosis, which is essential for the prompt initiation of appropriate therapy. The lack of significant clinical benefit from a regimen of mycophenolate mofetil, prednisone acetate, and irbesartan in this patient with concurrent AS and IgAN highlights the necessity for targeted diagnostics and personalized treatment strategies.

## Data Availability

The original contributions presented in the study are included in the article/supplementary material, further inquiries can be directed to the corresponding author/s.
